# Spouses and Depressive Symptoms in Older Adulthood

**DOI:** 10.1038/srep08594

**Published:** 2015-02-26

**Authors:** Neeti Pradeep, Angelina R. Sutin

**Affiliations:** 1 Florida State University College of Medicine

## Abstract

Depressive symptoms may co-occur within couples and follow similar trajectories, but relatively little is known about this process in old age. This study thus examined the association between some spousal characteristics (spouse's depressive symptoms, age difference between spouses) and the trajectory of depressive symptoms in older adults. Participants ≥65 years old were drawn from the Health and Retirement Study (*N* = 12,010; Mean age = 70.60 and 69.16 for target husbands and wives, respectively). Depressive symptoms were measured with a short form of the Center for Epidemiological Studies Depression (CES-D) scale. Hierarchical Linear Modeling was used to model up to 9 assessments of depressive symptoms of target spouses (Mean number of CESD assessments per target spouse = 3, range 1–9). Depressive symptoms between spouses were correlated; convergence over time was modest. For both husbands and wives, having a younger spouse was associated with more depressive symptoms at age 65. These results suggest that there is concordance between spouses' depressive symptoms and that the age difference between spouses contribute to depressive symptoms as couples enter old age. The association between spouses' depressive symptoms is nearly as strong as the effect of each decade increase in age.

Depression is a widespread mental disorder prevalent in later life. Even symptoms of depression that do not meet the threshold for major depressive disorder contribute to morbidity and loss of function^1^. After a midlife decline, depressive symptoms tend to increase again starting in the mid-60s, an increase not solely attributable to disease, functional limitations, or proximity to death^2^. Most research on depressive symptomatology in old age has either focused on factors related to the individual^3^ or relationship transitions (e.g., divorce, bereavement)^4^; less research has explored how older couples' depressive symptoms change together with age.

While spouses often offer emotional support that buffers against the stresses of aging, spouses may also have deleterious effects on each other. And, indeed, there tends to be a modest correlation between spouses' level of depressive symptoms concurrently and over time^5^. That is, as one spouse increases in depressive symptoms, so does the other spouse. Much of the research on spousal similarity, however, has been conducted on young and middle-aged adults; less research has focused on older adults.

Spouses can be similar or different on a number of characteristics. One of the most fundamental characteristics is the age difference (or similarity) between spouses. Yet, relatively little research has addressed the consequences of an age gap between spouses. Most of the research in this area has focused on the differential effects of an age gap on survival. Men married to younger wives, for example, tend to live longer, whereas women married to younger husbands tend to die younger^6^. This difference may be due, in part, to selection (i.e., healthier older men attract younger women)^6^. In addition to survival, an age gap between the spouses is also associated with mental health during widowhood. Among widows, for example, those who have a greater age gap with their deceased spouse have worse mental health than those who are of a similar age, perhaps due to the worse health and a greater sedentary lifestyle for couples in age heterogeneous marriages^7^. Less research has addressed the mental health correlates of age differences between living spouses in older adulthood. We extend this line of research to examine whether an age gap between spouses has a similar association with depressive symptoms during life.

The present research uses a large sample from a longitudinal study to examine how spouses' contribute to their partners' depressive symptoms in two ways. First, we test for longitudinal associations between spouses' depressive symptoms across older adulthood. Given that previous research has found modest cross-sectional correlations between spouses' depressive symptoms and that couples tend to show the greatest convergence before marriage^5^, we expect spouses' depressive symptoms to be correlated but that convergence between spouses over time will be modest in old age. Second, we test whether the age gap between spouses is associated with depressive symptoms and the trajectory of these symptoms over time. Given that age differences between spouses are associated with worse mental health in widowhood, we expect that an age gap between spouses will be associated with more depressive symptoms.

## Methods

### Participants

Participants were drawn from the Health and Retirement Study (HRS), a nationally-representative longitudinal study of Americans age 50 and older and their spouses^8^. We selected intact heterosexual couples up until the couple dissolved (through separation, divorce, or death) who completed at least one assessment between 1993 and 2010. Target participants were selected if they were over the age of 65; spouses of target participants, however, were retained even if they were younger than 65. Thus, there was not an even split in sex because spouses under the age of 65 were included in the analyses for the target group, but spouses under the age of 65 were not included as targets. We focus on target spouses 65 years and older because there is a linear increase in depressive symptoms after the age of 65, whereas there is a curvilinear effect up to age 65^2^. From the total HRS sample, there were 6582 target husbands and 5428 target wives over the age of 65. See Table 1 for demographic characteristics of the sample. The study was carried out in accordance with the Declaration of Helsinki. The Institutional Review Board (IRB) at the University of Michigan approved the methods and data collection in the HRS, and all participants provided informed consent before participating. In addition, the IRB at the Florida State University also approved analysis of these public data.

### Depressive Symptoms

Depressive symptoms were measured with a short version of the Center for Epidemiological Studies Depression scale^9^ that has been shown to be a reliable and valid short measure of depressive symptoms^10^. Since 1993, HRS participants rated nine items (yes/no) that measured negative (e.g., felt sad) and positive (e.g., felt happy) affect within the last week. The present research focused on the sum of the six negative affect items. We focus on the negative affect items because previous research has indicated that negative and positive affect do not change in similar ways with aging and that combining them can obscure age-related changes^2^,^11^. Both target husbands and target wives had an average of three CES-D assessments (Mean = 3.62 and 3.37 for husbands and wives, respectively; range 1 to 9). See Table 1 for descriptive statistics for the CESD.

### Statistical Approach

We estimated the trajectory of depressive symptoms using Hierarchical Linear Modeling (HLM) from up to nine assessments of depressive symptoms. HLM is a flexible approach that can be used to model growth trajectories over time^12^. In HLM analyses, the number of assessments and the spacing between assessments may vary across persons, given that the time-series observations for each participant are used to estimate each individual's trajectory (Level 1), and those individual parameters are the basis of group estimates (Level 2). Individuals who only participated on one occasion can still be used to help stabilize estimates, which allows all available data to be used in the analyses. This is a significant advantage of the HLM framework; missing data and varying timing tend to pose major problems in conventional approaches, such as repeated measures analyses of variance (ANOVA). In addition, longitudinal HLM can estimate age trajectories over a longer age span with data collected in a relatively shorter time interval. As such, HLM is a powerful analytic tool for testing change over time.

At Level 1, we fit a linear model to capture the increase in depressive symptoms in older adulthood. Age was centered at 65 years old so that the intercept represents the average number of depressive symptoms at age 65. We included spouses' concurrent depressive symptoms and the interaction between spouses' symptoms and target age as time-varying covariates at Level 1. These parameters estimate the average concurrent effect of spouses' depressive symptoms and whether this effect changes with age, respectively. We controlled for race (dummy coded as white/other = 0 Black = 1 and white/Black = 0 other = 1) and education (years of education centered at the mean for men and women) at Level 2. The equations for the models were:

Level 1: Target Spouse Depressive Symptoms = *γ*_0_ + *γ*_1_ (Age) + *γ*_2_ (Spouses' Depressive Symptoms) + *γ*_3_ (Spouses' Depressive Symptoms × Age) + *e*

Level 2: *γ*_0_ = *β*_00_ + *β*_01_ (Race [Black]) + *β*_02_ (Race [Other]) + *β*_03_ (Education)

*γ*_1_ = *β*_10_ + *β*_11_ (Race [Black]) + *β*_12_ (Race [Other]) + *β*_13_ (Education)

To examine whether age differences between spouses were associated with depressive symptoms, we included two additional dummy coded variables at Level 2 that contrasted younger and older spouses by at least 10 years with spouses closer in age. Specifically, we recoded age differences into a dummy variable for spouses that were at least 10 years older than the target spouse and another dummy-coded variable for spouses that were at least 10 years younger than the target spouse, compared to spouses that were within 10 years of each other. Analyses were run separately for target husbands and target wives.

We did several supplemental analyses to test whether the main findings could be due to other aspects of the spousal relationship. Specifically, in addition to the target's race and education, we controlled for number of previous marriages, number of children, educational differences between the spouses, and whether the marriage dissolved (vs. remained intact).

## Results

Consistent with previous research, depressive symptoms increased across older adulthood for both husbands and wives (Table 2). At age 65, wives reported significantly more depressive symptoms than husbands (*t* = 10.25, *p* < .01). The rate of increase in depressive symptoms was higher for wives, but not statistically different from that of husbands (*t* = 1.11, *ns*). Also consistent with previous research, African Americans and less educated participants reported more depressive symptoms than whites and more educated participants, respectively; there were no differences in the rate of change. These effects of race and education were similar for both target husbands and wives.

We next addressed our first question of interest, specifically whether spouses' depressive symptoms were associated with the intercept and slope of the target spouse (Table 2). For both husbands and wives, there were significant effects on the average level of depressive symptoms, which indicated a general correspondence between spouses' depressive symptoms. In other words, if the wives experienced depressive symptoms, so did the husbands, and vice versa. In terms of effect size, this association was about half the size of the effect of race on the intercept of depressive symptoms, but it was approximately twice as large as the effect of education. Further, it corresponded to roughly the effect of one decade of life. There was a small positive effect of spouses' depressive symptoms on rate of change for both husbands and wives: as wives increased in depressive symptoms, so did their husbands and vice versa. This effect, however, was modest.

Supplemental analyses revealed that, for both husbands and wives, the number of times married was unrelated to the intercept of depressive symptoms. The number of previous marriages was associated positively with the slope of depressive symptoms for wives (β = .13, SE = .04, *p* < .01), but not for husbands (β = .04, SE = .03, *ns*). Having children was associated positively with depressive symptoms at age 65 (β = .02, SE = .01, *p* < .05) for husbands but was unrelated to the intercept for wives (β = .01, SE = .01, *ns*). For both spouses, having children was associated with less of an increase in depressive symptoms in old age (β for both husbands and wives = −.02, SE = .01, *p* < .05). Finally, educational differences between the spouses and whether the marriage dissolved were both unrelated to either the intercept or slope of depressive symptoms for either spouse. Including previous marriages, children, educational differences, and whether the marriage dissolved did not alter the results from the main analyses, which indicated that these factors could not account for the relation between spousal depressive symptoms.

The second question we addressed was whether an age difference between the spouses was associated with depressive symptoms (Table 2). For both husbands and wives, a greater age difference with his/her spouse was associated with a higher average level of depressive symptoms, controlling for race and education. Specifically, for both husbands and wives, marriage to someone at least 10 years younger was associated with a higher level of depressive symptoms. Marriage to an older spouse was associated with less of an increase in depressive symptoms for husbands; age differences were unrelated to change in depressive symptoms for wives. Controlling for number of previous marriages, children, differences in education between spouses, and whether the marriage dissolved did not account for these associations; the results were identical when these factors were included in the analyses.

## Discussion

The present research examined how spousal characteristics are associated with depressive symptoms in older adulthood. We found a concordance between husbands' and wives' depressive symptoms: If husbands were experiencing depressive symptoms, so were their wives, and vice versa. Although modest, this concordance grew stronger with age. In addition, age differences between spouses were also associated with more depressive symptoms, with both husbands and wives married to younger spouses reporting more depressive symptoms at age 65 than spouses closer in age. Race, education (either the target spouse's own education or educational differences between the spouses), number of previous marriages, and children could not account for any of the reported findings.

There are a number of reasons why spouses' depressive symptoms may be similar, such as non-random mating and convergence. Non-random mating suggests that individuals choose partners similar to themselves by picking a mate based on similar phenotypes (i.e., assortative mating) and/or similar social backgrounds (i.e., social homogamy). Convergence, in contrast, may be due to a shared environment (e.g., financial pressures, shared health-risk behaviors, illness) and/or emotional contagion (i.e., depressive symptoms may spread from one spouse to the other). Although there is evidence for non-random mating, convergence appears to play a stronger role in spousal similarity for psychological distress^5^. The present research indicates a similar concordance of depressive symptoms as in studies of younger adults. We could not, however, tease apart whether the similarity was due to non-random mating or to convergence, and we found only very modest evidence that spouses' depressive symptoms change together in older adulthood.

Our findings contribute to understanding the pattern of spousal depressive symptoms across the lifespan. In young adulthood, the convergence between spouses for mental health-related variables tends to be highest in the years just before entering into marriage^5^. Interestingly, after an initial convergence across the early years of marriage in young adulthood, the correlation between spouses' mental health tends to diverge but converges again in middle adulthood^5^. A study of middle-aged spouses likewise found modest convergence between spouses depressive symptoms across middle adulthood and that there was a stronger convergence for wives than for husbands^13^. Our results dovetail nicely with the findings of these two studies in that spouses continue the midlife convergence into old age (albeit modestly) and that the average correlation between depressive symptoms is somewhat (although not significantly) stronger for wives than for husbands.

In addition to concordance, the age difference between spouses was also associated with depressive symptoms. Husbands and wives with younger spouses reported more depressive symptoms at age 65. For husbands, age differences were also associated with less increase in depressive symptoms in old age; there was no similar association for wives. A number of processes, including social comparison between the younger and older spouse, differences in the actual rate of aging between spouses, and stereotypes of aging may contribute to these associations. For example, as older spouses enter old age, they may compare themselves (e.g., their physical and cognitive health) to their younger spouses and any perceived differences may contribute to feeling more general negative affect. Indeed, these factors have been hypothesized to contribute to the effect of spousal age differences on differential physical health outcomes^14^. In addition, spousal age differences have been related to adverse mental health following the death of the spouse^7^. The present research suggests that such age differences also extend to depressive symptoms among living spouses.

This research had several strengths, including a large sample of spouses and the longitudinal design. Future research could address the reasons for concordance in older adulthood, why spouses of different ages report more depressive symptoms, whether the effects found here extend to same-sex couples, and whether the effects are similar for clinical depression, not just negative affect. The present research, however, is a step toward understanding how spouses contribute to depressive symptoms in older adulthood. Our results show, for example, that there is a relation between spouses' depressive symptoms when measured at the same time and that there is a modest convergence over time. The concurrent relation was much stronger than the convergence, which suggests that the emotional dynamics within the couple may be playing out on a more immediate time scale than HRS captured with its bi-annual assessments. More frequent assessments are thus needed to address critical questions of emotional contagion.

Depressive symptoms have a strong interpersonal component, and such relations reflect the complexity of human life and human relationships. Indeed, close relationships can have profound psychological effects on the people involved. Caregivers, for example, are more distressed than non-caregivers^15^, and individuals who care long-term for their spouses are at higher risk for severe depressive symptoms^16^. A narrow focus on intra-individual factors that increase risk for depression may miss important factors that contribute to distress. When treating older adults, the social context in which the individual lives, including their spouse, needs to be addressed.

## Author Contributions

N.P. conceived of the project and conducted the analyses. N.P. and A.R.S. wrote and reviewed the main manuscript text.

## Figures and Tables

**Table 1:** Descriptive Statistics for All Study Variables

	Target Spouse	
Demographic factor	Husbands	Wives
Age (First Assessment)	70.06 (5.84)	69.16 (5.02)
Race (Black)	10.9%	9.5%
Race (Other)	2.2%	1.7%
Education	12.01 (3.65)	12.10 (3.01)
# CESD Assessments	3.62 (2.25)	3.37 (2.11)
CESD Score (first assessment)	.92 (1.33)	1.18 (1.54)
CESD Score (most recent)	1.06 (1.44)	1.33 (1.62)
Absolute age difference between spouses	4.94 (4.87)	3.94 (3.69)

*Note*. *N* = 6,582 for target husbands and *n* = 5,428 for target wives. Table presents means (standard deviations) or percentages.

**Table 2:** Estimated Trajectory of Depressive Symptoms in Older Adulthood

	Target Spouse	
	Husbands' DS	Wives' DS
Level 1		
Intercept	.62 (.02)*	.91 (.02)*
Slope	.14 (.02)*	.18 (.03)*
Spouse DS	.12 (.01)*	.16 (.02)*
Spouse DS × Age	.02 (.01)*	.03 (.01)*
Level 2 Intercept		
Race (Black)	.25 (.06)*	.24 (.08)*
Race (Other)	.25 (.12)*	.04 (.17)
Education	−.06 (.01)*	−.11 (.01)*
Younger Spouse	.20 (.06)**	.47 (.18)**
Older Spouse	.24 (.27)	.08 (.09)
Level 2 Slope		
Race (Black)	−.01 (.06)	−.11 (.09)
Race (Other)	−.20 (.13)	.12 (.21)
Education	.00 (.00)	.00 (.01)
Younger Spouse	−.06 (.04)	−.14 (.14)
Older Spouse	−.76 (.32)*	.07 (.11)

*Note*. *n* = 6582 for target husbands and *n* = 5428 for target wives. Coefficients are estimates (standard errors) from Hierarchical Linear Modeling analysis. DS = Depressive Symptoms.

^*^p < .05.

^**^p < .01.
